# Supplementary material for: PD-L1 and AKT Overexpressing Adipose-Derived Mesenchymal Stem Cells Enhance Myocardial Protection by Upregulating CD25+ T Cells in Acute Myocardial Infarction Rat Model
Source: Int J Mol Sci. 2023 Dec 21;25(1):134. doi: 10.3390/ijms25010134 (PMC10779305; doi:10.3390/ijms25010134)
Supplement: Supplementary file 1 [file ijms-25-00134-s001.zip › ijms-2733528-supplementary.pdf]

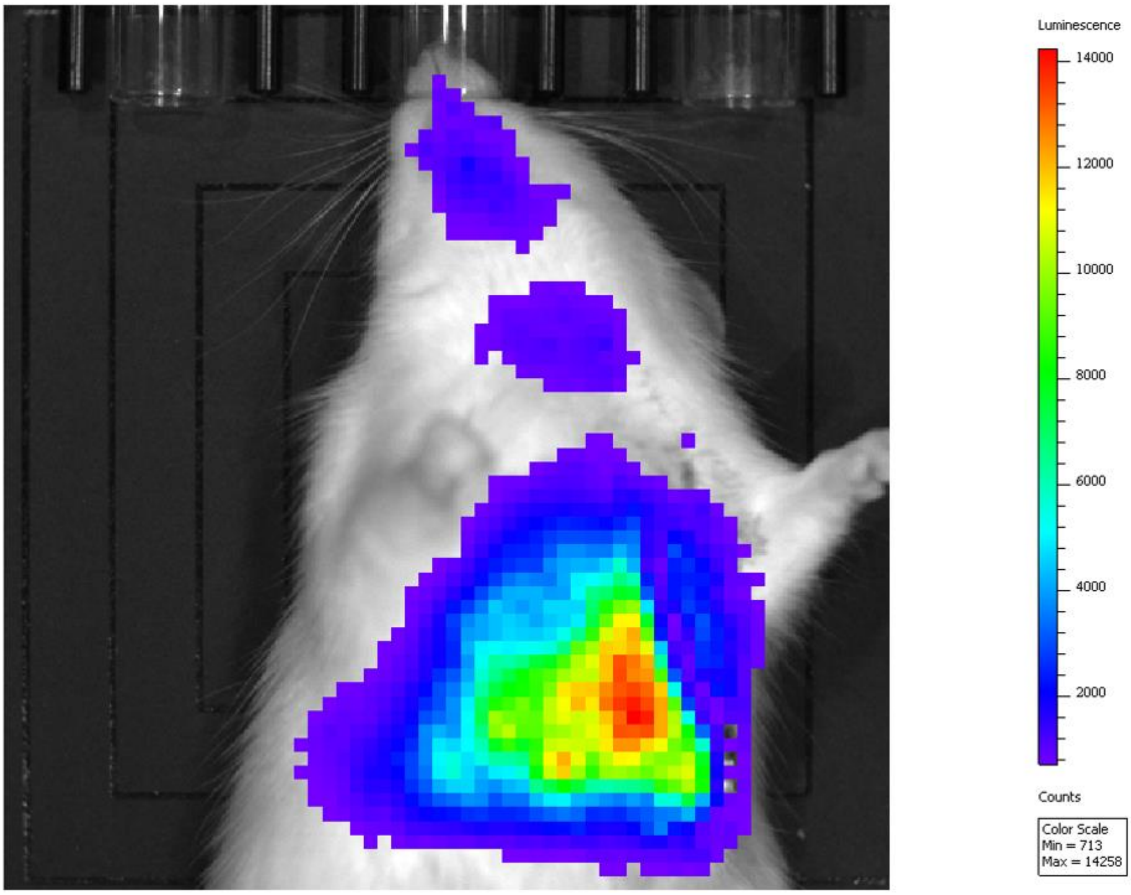

Figure S1: Representative images acquired by an in vivo imaging system (IVIS Lumina XRMS System, PerkinElmer, Waltham, MA, USA) showing the D-luciferin bioluminescence imaging of a single representative rate from ADMSC-PDL1-Akt group.
